# Cerebellar mass as a primary presentation of papillary thyroid carcinoma: case report and literature review

**DOI:** 10.1186/1758-3284-1-23

**Published:** 2009-06-29

**Authors:** Saleh Fahad Al-Dhahri, Abdullah Sulieman Al-Amro, Wafa Al-Shakwer, Abdullah Sulieman Terkawi

**Affiliations:** 1 Head & Neck department, King Fahad Medical City, Riyadh, Saudi Arabia; 2 Radiation Oncology department, King Fahad Medical City, Riyadh, Saudi Arabia; 3 Pathology department, King Fahad Medical City, Riyadh, Saudi Arabia

## Abstract

**Background:**

Papillary carcinoma is the most common differentiated malignant thyroid neoplasm. The biological course of this cancer is typically indolent with a protracted clinical course. Metastases commonly occur in regional lymph nodes, and distant metastasis is a late and rare occurrence. We report a patient who presented with cerebellar metastasis prior to the diagnosis of papillary thyroid carcinoma and review the literature of brain metastasis from papillary thyroid carcinoma.

**Results:**

A 75-year old female presented at the emergency room with progressive dizziness, headache and vomiting, where a brain CT and MRI showed a posterior cerebellar tumor. Surgical resection revealed papillary carcinoma consistent with thyroid origin. Subsequent ultrasound and CT-scan revealed a thyroid nodule, after which the patient underwent total thyroidectomy. Pathologic evaluation was consistent with papillary thyroid carcinoma.

**Conclusion:**

Brain metastasis may rarely be the initial presentation of papillary thyroid carcinoma. Solitary brain metastasis can completely be resected with better prognosis.

## Introduction

Papillary thyroid carcinoma (PTC) is the most common thyroid cancer, representing approximately 80% to 90% of all newly diagnosed thyroid cancers [1]. PTC is typically characterized by an indolent clinical course in comparison to other differentiated and undifferentiated thyroid malignancies [2,3]. Radiation is the best known risk factor for PTC [2].

PTC commonly metastasizes to lymph nodes but [1] distant metastases at time of presentation may rarely occur and account for 9% to 10% during the course of follow-up [2]. The presence of distant metastases is a grave event associated with decreased survival rates of 37% and 24% at 5 and 10 years respectively [4]. Brain metastasis from a thyroid primary is extremely rare with approximately 23 reported instances [5-14].

We reported a patient who presented initially with cerebellar metastasis from an undiagnosed primary PTC and review reported instances of PTC with brain metastasis.

## Case presentation

A 75-year old female presented at the emergency room with dizziness, headache and vomiting for five days. Physical examination was unremarkable except for hoarseness of voice of one-year duration, which was investigated with negative physical findings.

**Figure 1:**
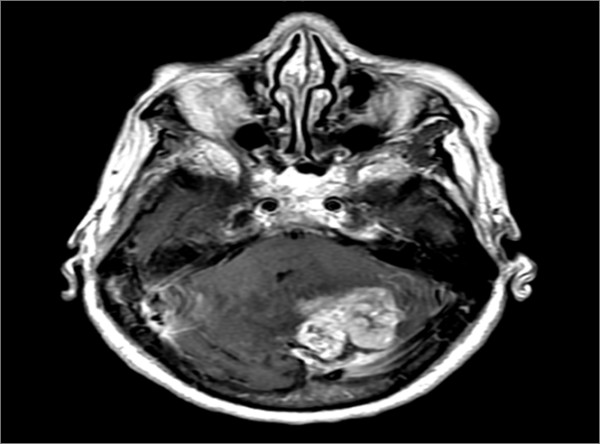
**Brain MRI showed hemorrhagic mass involving the medial aspect of the left cerebellum and crossing the midline to the right hemisphere**.

She was referred to the neurological service where a computerized tomography (CT) of the brain showed a left posterior fossa mass of high density, causing compression on the basal cistern and obstructive hydrocephalus. Brain Magnetic Resonance Imaging (MRI) showed a hemorrhagic mass lesion involving the medial aspect of the left cerebellum (Figure 1).

The patient underwent a left occipital craniotomy and excision of the cerebellar tumor that showed metastasis consistent with thyroid primary.

**Figure 2:**
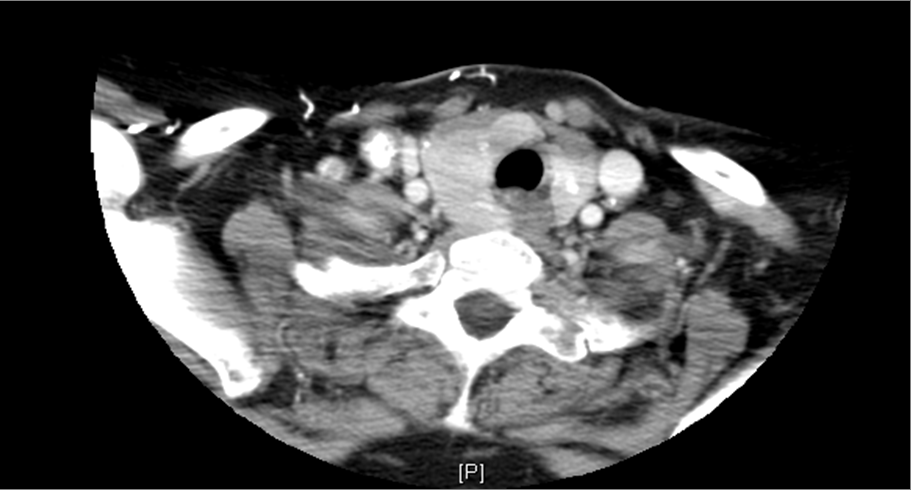
**A neck CT scan with contrast of the patient showing a right thyroid lesion**.

A subsequent thorough neck examination revealed a fairly palpable right thyroid lobe nodule and small right-neck lymph nodes. A neck ultrasound (US) confirmed the presence of an ill-defined nodule located posteriorly in the right thyroid lobe. A follow-up neck CT with contrast (Figure 2) showed enlarged homogeneous thyroid gland with multiple calcifications; only one calcified lymph node could be identified in the right jugulodigastric chain. A US-guided fine needle aspiration from the right lobe was performed and rendered a diagnosis suggestive of papillary thyroid carcinoma. The patient underwent total thyroidectomy, during which an involvement of the right recurrent laryngeal nerve was noted and right lymph nodes neck dissection. The postoperative course was uneventful, and the patient was treated with radioiodine.

### Pathology

#### Brain Resection

##### Gross findings

Brain specimen consisted of fragments of friable light tan and soft tumor tissue admixed with brain tissue.

##### Histopathology

**Figure 3:**
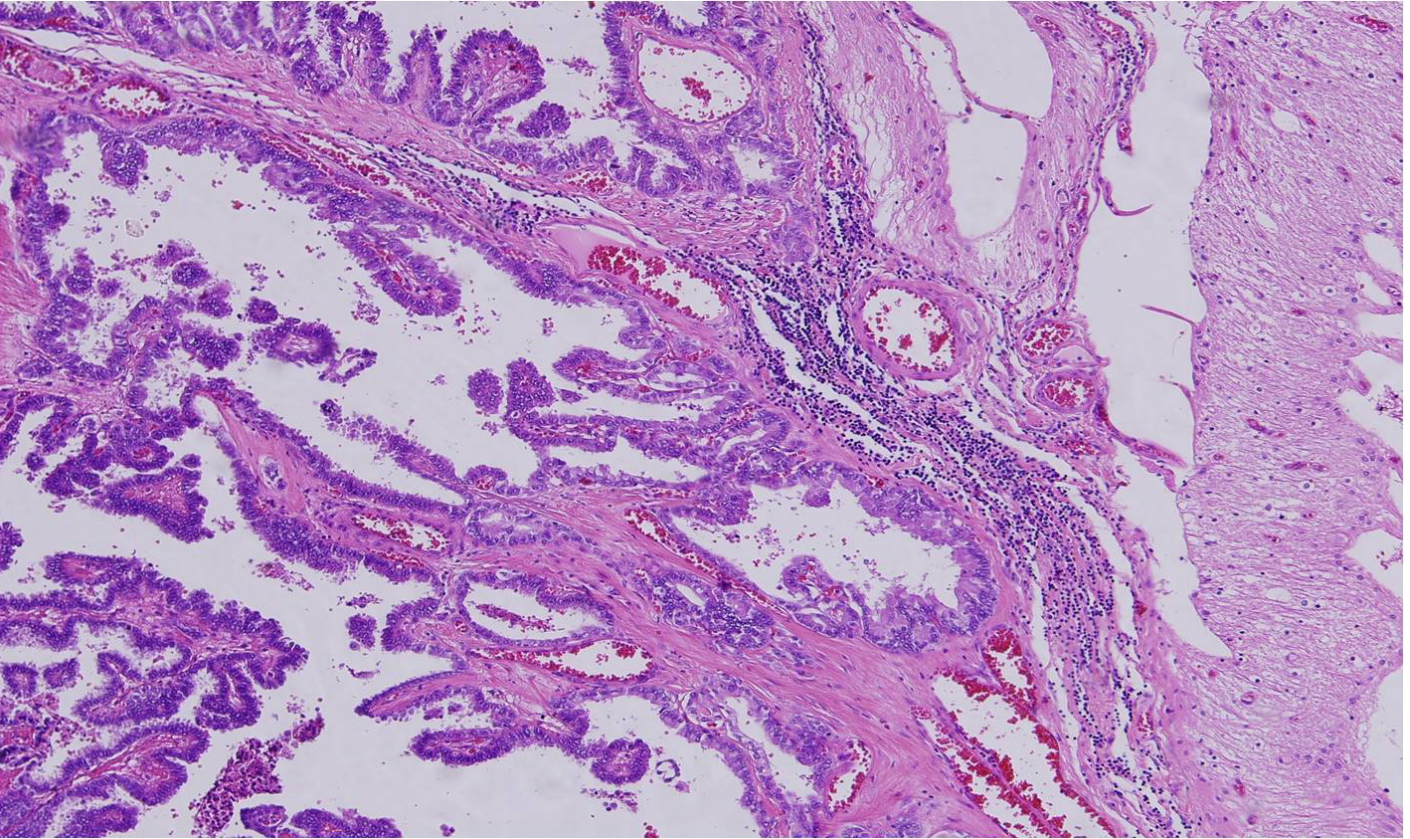
**Photomicrograph of the brain metastasis showing papillary carcinoma consistent with thyroid primary**.

Tumor showed papillary fronds with fibrovascular core lined by columnar-to-cuboidal tumor cells, with occasional nuclear inclusion and grooving and abundant cytoplasms consistent with metastatic papillary thyroid carcinoma (Figure 3).

##### Fine Needle Aspiration

The aspirated materials comprised of individual cells and cluster of tumor with fibro-vascular core. The tumor cells exhibited nuclear grooving and intracellular inclusion bodies consistent with papillary thyroid carcinoma.

#### Thyroid Resection

##### Gross findings

Gross examination of the thyroid revealed a well-circumscribed, light-tan and friable 2.5-cm nodule in the right lobe.

##### Histopathology

**Figure 4:**
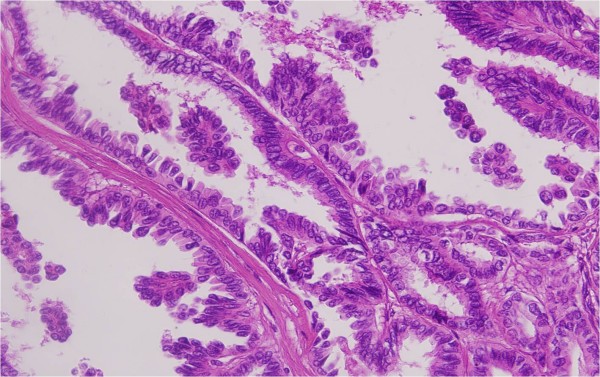
**Photomicrograph of the primary thyroid carcinoma illustrating papillary carcinoma with tall cell features**.

The tumor manifested papillary formation lined by columnar cells with clear and oncocytic cytoplasmic features and nuclear characteristics consistent with papillary thyroid carcinoma, tall cell variant. Of the 13 lymph nodes dissected, only one was positive for papillary carcinoma (Figure 4). The thyroid tumor showed identical features with the brain lesion.

##### Immunohistochemically

**Figure 5:**
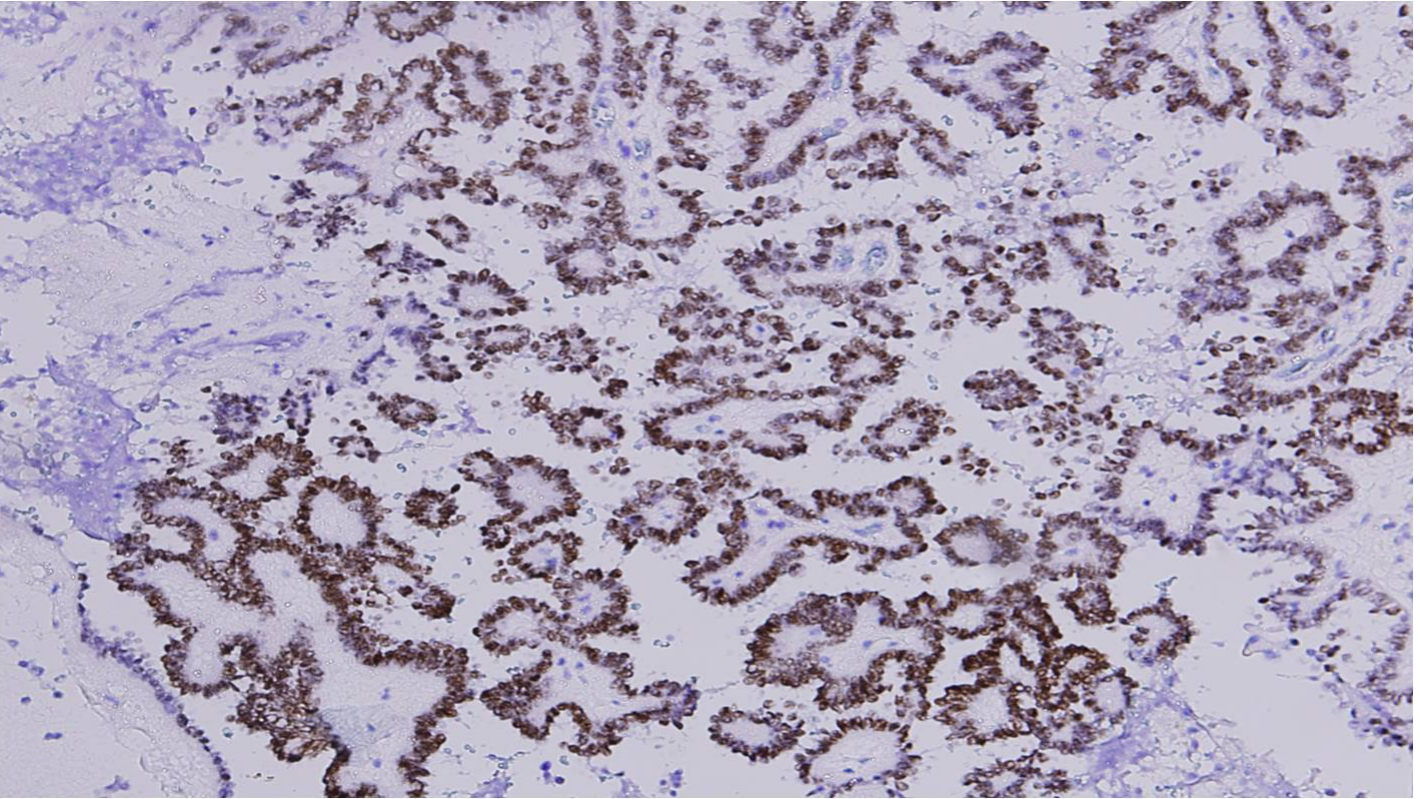
**A thyroid transcriptional factor immunostaining of the brain metastasis revealing strong nuclear staining in tumor cells**.

The tumor cells reacted to epithelial membrane antigen, cytokeratin, thyroid transcription factor (TTF1) (Figure 5) and focally to thyroglobulin antibodies.

## Discussion

We present, to our knowledge, the first instance of cerebellar metastasis of papillary thyroid carcinoma prior to the identification of primary thyroid papillary carcinoma. Prior cases of brain metastasis from occult carcinoma measuring (< 3 mm) were recently published [12,13]. In this report, the patient presented with neurological symptoms initially, but the thyroid primary was identified at the time [12,13]. In both of these reports, the site of the metastasis was the occipital lobes. Brain metastases are rare (0.15 – 1.3% of all metastatic thyroid carcinoma) [4]. Although several brain metastasis have been reported, (Additional file 1) the primary thyroid carcinoma was diagnosed at the time of the brain metastasis [5-11,14,15].

The most common sites for distant metastasis of PTC are the lungs, followed by the bone [3], and rarely to the skin, liver and brain [2-17]. The literature review showed a reported case of metastasis to each of the following sites: breast, parotid, adrenal, pituitary, kidney, porta hepatis, the orbit, the sphenoid sinus, pancreas and the skeletal muscles.

Several investigators have attempted to determine the risk factors for distant metastases which include male gender, advanced age, histologic grade, extrathyroidal invasion at initial examination, and completeness of surgical resection of the primary tumor [1,16].

Chiu A C et al, [18] reported 47 instances of brain metastases from thyroid cancer (32 of them with differentiated carcinoma), that were presented at M.D Anderson Cancer Center between March 1944 through September 1995. In that study, 68% were identified during the subsequent course of disease, 23% discovered at autopsy, and only 15% as a primary clinical feature at initial presentation. The median survival was 4.7 months once the brain metastases were diagnosed, with a disease-specific mortality rate of 78%. A solitary lesion with complete surgical resection was found to be associated with a better prognosis.

Misaki T et al [17] reported nine cases of differentiated thyroid carcinoma that metastasized to the brain; seven of them were PTC. They noticed that the median survival time for patients after discovery of brain metastases is 9.4 months. None of the brain lesions showed significant uptake of radioactive iodine, probably due to the blood brain barrier. It was recommended that CT scan or MRI should be performed for any patient with suspicious neurological symptoms. For therapy they recommended radiosurgery as alternative for surgical removal, although they used it for only 2 patients.

Radioiodine scan may not be reliable to detect brain metastasis from PTC [16,17]; however, a high serum thyroglobulin level may be helpful but not specific [16,17], as it is usually high in all metastatic disease.

Surgery, radiotherapy, and radioactive iodine therapy have been used with varying results for treatment of brain metastases from papillary thyroid carcinoma. The best therapeutic option seems to be resection, followed by radioactive iodine therapy [4], although there is no clearly defined protocol concerning the management of intracranial metastases [4].

Retrospectively, the cause of the patient's hoarseness of voice was the invasion of the recurrent laryngeal nerve by the PTC. Approaching a patient with brain metastases should involve the thyroid gland and the possibility of brain metastases from PTC during the course of follow-up should be kept in mind.

## Conclusion

• Brain metastases secondary to PTC are rare and its presentation before diagnosis of primary tumor is only reported once, with our case. Due to its rarity, insufficient data are present in its course, prognosis, mortality, and management.

• There has been a general consensus in the literature that brain metastases are associated with poor prognosis with tendency for recurrence, so close follow-up is mandatory.

• Surgery is the treatment of choice for solitary resectable brain tumor, and radiotherapy should preserve for patients with multiple lesions, or unresectable tumors. Although this field still need more studies.

• Thyroid imaging can be helpful as a part of diagnostic workup for patients with vocal cord paralysis and brain metastasis.

## Competing interests

The authors declare that they have no competing interests.

## Authors' contributions

SFA-D:the primary thyroid surgeon and writer. ASA-A:management and writer. WA-S:pathological diagnosis and writer. ASA-T:writer, alignment and drafted the manuscript.

## Consent

A written informed consent was obtained from the patient for publication of this case report and accompanying images.

## Supplementary Material

Additional file 1 **Supplementary table**. Clinicopathologic findings of reported PTC with Brain metastasis Click here for file

## References

[B1] Boone RT, Fan CY, Hanna EY (2003). Well-differentiated carcinoma of the thyroid. Otolaryngol Clin North Am.

[B2] Tuttle RM, Leboeuf R, Martorella AJ (2007). Papillary thyroid cancer: monitoring and therapy. Endocrinol Metab Clin North Am.

[B3] Brunicardi FC, Andersen DK, Billiar TR, Dunn DL, Hunter JG, Matthews JB, Pollock RE, Schwartz SI (2005). Schwartz's Principles Of Surgery.

[B4] Pazaitou-Panayiotou K, Kaprara A, Chrisoulidou A, Boudina M, Georgiou E, Patakiouta F, Drimonitis A, Vainas I (2005). Cerebellar metastasis as first metastasis from papillary thyroid carcinoma. Endocr J.

[B5] Aguiar PH, Agner C, Tavares FR, Yamaguchi N (2001). Unusual brain metastases from papillary thyroid carcinoma: case report. Neurosurgery.

[B6] Aihara N, Nagai H, Mase M, Shimazu N, Kanai H, Kamiya K (1991). Brain metastasis of thyroid papillary carcinoma – case report. Neurol Med Chir (Tokyo).

[B7] Goolden AW, McLaughlin JE, Valentine AR, Pease C (1990). Solitary cerebral metastasis from a papillary carcinoma of the thyroid. Postgrad Med J.

[B8] Ibanez ML, Russell WO, Albores-Saavedra J, Lampertico P, White EC, Clark RL (1966). Thyroid carcinoma – biologic behavior and mortality. Postmortem findings in 42 cases, including 27 in which the disease was fatal. Cancer.

[B9] Jyothirmayi R, Edison J, Nayar PP, Nair MK, Rajan B (1995). Case report: brain metastases from papillary carcinoma thyroid. Br J Radiol.

[B10] Kapusta LR, Taylor M, Ang LC, Schwartz M (1999). Cytologic diagnosis of a solitary brain metastasis from papillary carcinoma of the thyroid. A case report. Acta Cytol.

[B11] Maruyama M, Kobayashi S, Shingu K, Nagashima H, Nagamine K, Kasuga Y, Kato R, Kameko F, Amano J (2000). Solitary brain metastasis from papillary thyroid carcinoma in a patient with depression: report of a case. Surg Today.

[B12] Michie HR, O'Bryan-Tear CG, Marsh H, Glazer G (1987). Cerebral metastases from occult papillary carcinoma of the thyroid. Br J Surg.

[B13] Ota T, Bando Y, Hirai M, Tanaka N, Takabatake Y, Kasahara Y, Fujisawa M (2001). Papillary carcinoma of the thyroid with distant metastases to the cerebrum: a case report. Jpn J Clin Oncol.

[B14] Pacak K, Sweeney DC, Wartofsky L, Mark AS, Punja U, Azzam CJ, Burman KD (1998). Solitary cerebellar metastasis from papillary thyroid carcinoma: a case report. Thyroid.

[B15] Parker LN, Wu SY, Kim DD, Kollin J, Prasasvinichai S (1986). Recurrence of papillary thyroid carcinoma presenting as a focal neurologic deficit. Arch Intern Med.

[B16] Cha ST, Jarrahy R, Mathiesen RA, Suh R, Shahinian HK (2000). Cerebellopontine angle metastasis from papillary carcinoma of the thyroid: case report and literature review. Surg Neurol.

[B17] Misaki T, Iwata M, Kasagi K, Konishi J (2000). Brain metastasis from differentiated thyroid cancer in patients treated with radioiodine for bone and lung lesions. Ann Nucl Med.

[B18] Chiu AC, Delpassand ES, Sherman SI (1997). Prognosis and treatment of brain metastases in thyroid carcinoma. J Clin Endocrinol Metab.

