# Supplementary material for: Cerebellar mass as a primary presentation of papillary thyroid carcinoma: case report and literature review
Source: Head Neck Oncol. 2009 Jun 29;1:23. doi: 10.1186/1758-3284-1-23 (PMC2712461; doi:10.1186/1758-3284-1-23)
Supplement: Additional file 1 — Supplementary table. Clinicopathologic findings of reported PTC with Brain metastasis [file 1758-3284-1-23-S1.doc]

| **Table 1. Clinicopathologic findings of reported PTC with Brain metastasis** | | | | | | | | | | | | | | |
| --- | --- | --- | --- | --- | --- | --- | --- | --- | --- | --- | --- | --- | --- | --- |
| ***Author/published year/***  ***reference number*** | ***Number***  ***of cases*** | ***Age (years)***  ***Gender*** | ***Initial presentation*** | ***Location within the brain*** |  | ***Interval from initial diagnosis*** | | ***Cervical lymph-adenopathy*** | | ***Other distant metastases*** | | ***Therapy for brain metastasis*** | | ***Evolution after surgery*** |
| **Ibanez et al., (1966)** | 1 | 67/M | Neck mass | Rt parietal |  | 12 yr | Yes | | Lung, bone | | Surgery | | Immediate post-op death | |
| **Parker et al., (1986)** | 2 | 31/M  74/M | Neck mass  Neck mass | Rt fronto-  parietal  Rt temporo-parietal |  | 35 yr  4 yr | Yes  Yes | | Lung, bone  None | | Surgery + RAI  Surgery | | Well and alive  Brain recurrence after 4 months | |
| **Michie et al., (1987)** | 1 | 47/M | Headache | Lt occipital  Rt frontal* |  | N/A | No | | None | | Surgery  RT* | | Brain recurrence after 6 months | |
| **Goolden et al., (1990)** | 1 | 52/F | Hoarseness | Rt frontal |  | 4 yr | No | | None | | RT | | Died after 6 months | |
| **Aihara et al., (1991)** | 1 | 55/F | Neck mass | Rt parietal |  | 15 yr | Yes | | Vertebra | | Surgery | | No recurrence for 1 yr | |
| **Jyothirmayi R et al., (1995)** | 2 | 51/M  59/M | Neck mass  Neck mass | Rt cerebellum  Rt frontal |  | 19 yr  2 yr | No  Yes | | Lung  None | | Surgery + RAI  Surgery + RAI | | Recurrence in bone  N/A | |
| **Pacak K et al., (1998)** | 1 | 82/F | Neck mass | Lt cerebellum |  | 8 yr | Yes | | Lung & vertebra | | Surgery | | No recurrence for 1 yr | |
| **Kapusta et al., (1999)** | 1 | 43/F | Neck mass | Rt frontal |  | 30 yr | Yes | | Lung | | RT | | N/A | |
| **Maruyama et al ., (2000)** | 1 | 52/M | Neck mass | Lt frontal |  | 8 yr | Yes | | Lung, bone | | Surgery | | Died due to AP | |
| **Cha ST et al., (2000)** | 1 | 78/F | Neck mass | Lt CP angle |  | 3 yr | N/A | | None | | Surgery, RT, EBR, GKRS | | Alive and well for 3 yr | |
| **Misaki T et al., (2000)** | 7 | 64/M  64/F  49/M  59/F  53/M  61/F  84/F | N/A  N/A  N/A  N/A  N/A  N/A  N/A | Lf frontal  Multiple  Multiple  Cerebellum  Lt parietal  Lt parietal  Cerebellum |  | 3 yr  1 yr  ½ yr  8 yr  6 yr  2 yr  9 yr | N/A  N/A  N/A  N/A  N/A  N/A  N/A | | Lung  Bone & lung  Lung  Lung  Bone & lung  Bone & scalp  Lung | | RT  RT  RT  Surgery  Surgery  SRS  SRS | | Died after 9 mo  Died after 6 mo  Died after 7 mo  Died after 23 mo  Died after 9 mo  Alive after 42 mo  Alive for 3 mo | |
| **Ota T et al., (2001)** | 1 | 53/M | Headache  & vision impairment | Rt parieto-occipital  & Rt frontal |  | Simultaneously | Yes | | None | | Surgery + RAI | | Regional lymphatic recurrence after 6 months | |
| **Aguiar et al., (2001)** | 1 | 33/F | Neck mass | CP angle and multiple in supra-tentorial space |  | 3 yr | N/A | | Lung | | Surgery & RT | | No recurrence in 35 months after resection | |
| **Pazaitou et al., (2005)** | 1 | 69/M | Neck mass | Rt cerebellum |  | 4 yr | No | | None | | Surgery + RT | | Died after 1 month | |
| **Our case** | 1 | 75/F | Dizziness | Lt cerebellum |  | N/A | Yes | | None | | Surgery | | Well and alive for 4 months | |
| **PTC: papillary thyroid carcinoma, RAI; radioactive iodine, U/S; ultrasound, RT; radiotherapy, CP; Cerebellopontine, EBR; external beam radiation, GKRS; gamma knife radiosurgery, AP; aspiration pneumonia. SRS; Stereotactic radiosurgery. *The second metastasis after 6 months.** | | | | | | | | | | | | | | |
